# Genetic Dissection of Image-Derived Pod-Related Traits in an Interspecific Soybean RIL Population

**DOI:** 10.3390/plants15182769

**Published:** 2026-09-10

**Authors:** Fangguo Chang, Tuanjie Zhao, Shunchang Su, Xiaohan Ruan, Liping Wei

**Affiliations:** 1 College of Agronomy, Gansu Agricultural University, Lanzhou 730070, China; 2 Key Laboratory of Biology and Genetics Improvement of Soybean, Ministry of Agriculture, Zhongshan Biological Breeding Laboratory (ZSBBL), National Innovation Platform for Soybean Breeding and Industry-Education Integration, State Key Laboratory of Crop Genetics & Germplasm Enhancement and Utilization, College of Agriculture, Nanjing Agricultural University, Nanjing 211800, China; 3 College of Information Science and Technology, Gansu Agricultural University, Lanzhou 730070, China

**Keywords:** soybean, deep learning, phenotyping, QTL, linkage mapping

## Abstract

Soybean pod-related traits are important agronomic characteristics associated with seed development, domestication, cultivar identification, and breeding improvement. However, conventional phenotyping methods mainly rely on manual measurements, which are time-consuming and labor-intensive and capture only limited dimensions of pod variation, while the genetic basis of skeleton- and curvature-based pod descriptors remains insufficiently characterized in biparental populations. In this study, eight quantitative traits representing pod size, shape, and color components were extracted from an existing mature pod image dataset of an interspecific soybean recombinant inbred line (RIL) population using the established deep learning-based image phenotyping framework. These traits exhibited substantial phenotypic variation, with across-year entry-mean broad-sense heritability *(H*^2^) estimates ranging from 0.30 to 0.80. Composite interval mapping (CIM) based on a high-density genetic linkage map identified 54 quantitative trait loci (QTLs), which were integrated into 39 non-redundant loci, including six cross-year stable QTLs and three QTLs supported by best linear unbiased prediction (BLUP) analysis. Candidate genes within selected focal QTL regions were prioritized through functional annotation and pod and seed developmental expression analyses. Among them, *Glyma.17G109100* (*GmSW17*) was prioritized as a positional candidate gene for pod size-related variation, whereas *Glyma.19G120400* (*L1*), a previously validated causal gene for pod color, was located within *qV19*. These findings demonstrate the effectiveness of combining deep learning-based phenotyping with genetic analysis for dissecting the genetic architecture of complex soybean pod-related traits and provide valuable stable QTLs and candidate genes for future functional studies and soybean molecular breeding.

## 1. Introduction

Soybean [*Glycine max* (L.) Merr.] is one of the most important legume crops worldwide, providing a major source of vegetable oil and plant protein for human consumption, animal feed, and industrial applications [1]. As the reproductive organ enclosing and protecting developing seeds, the pod plays indispensable roles in seed development, assimilate accumulation, and final yield formation. Pod-related traits, particularly pod morphology and pod color, are important agronomic characteristics widely used for cultivar description, germplasm identification, and breeding programs [2,3]. These traits have also undergone substantial changes during soybean domestication and subsequent genetic improvement, reflecting both natural and artificial selection for desirable agronomic characteristics [4,5]. Furthermore, pod growth and development are closely coordinated with seed growth and development, making pod-related traits informative indicators for evaluating seed-related characteristics and yield potential [6,7]. Therefore, accurate and systematic characterization of pod-related traits provides an important foundation for genetic studies and soybean breeding.

Accurate characterization of pod-related traits relies on reliable and quantitative phenotypic measurements. Traditionally, mature soybean pods have been evaluated using manual measurements and visual assessments, with overall size- and color-related characteristics serving as the primary descriptors for phenotypic evaluation [2]. Although these conventional approaches have contributed substantially to soybean genetic research and breeding practice, they remain time-consuming, labor-intensive, and difficult to apply in large-scale phenotyping [8,9]. More importantly, the major limitation of conventional phenotyping lies not only in measurement efficiency but also in the limited phenotypic information that can be captured. Existing descriptors mainly characterize overall pod size or visual appearance and are often insufficient to fully describe the complex morphological variation among mature soybean pods [9,10,11]. Consequently, subtle yet biologically meaningful morphological differences may remain undetected, restricting comprehensive phenotypic characterization and subsequent genetic dissection of pod-related traits. Thus, more informative quantitative descriptors are needed to achieve a deeper understanding of pod-related trait variation in soybean.

Recent advances in high-throughput phenotyping have greatly enhanced the efficiency and accuracy of quantitative trait evaluation in plants [12,13,14,15]. Among these technologies, image-based phenotyping has emerged as an effective approach for objective and non-destructive characterization of plant morphological traits [14,15,16,17]. The rapid development of deep learning has further improved the accuracy and robustness of image analysis, particularly in image segmentation and quantitative trait extraction under complex imaging conditions [8,9,10,16,18,19]. These approaches have been successfully applied to various crop species, including soybean, peanut, and other legumes, enabling rapid quantification of diverse quantitative traits associated with seed morphology, pod characteristics, and reproductive organ development [8,9,10,20,21]. Nevertheless, most previous studies have primarily focused on developing phenotyping pipelines or improving phenotyping efficiency, whereas relatively few have explored the integration of morphological descriptors with genetic analyses of complex quantitative traits [3,21]. Therefore, combining high-throughput phenotypic characterization with genetic mapping represents a promising strategy for dissecting the genetic basis of complex agronomic traits.

Pod-related traits are complex quantitative traits influenced by both genetic and environmental factors; however, their genetic dissection has received relatively limited attention compared with other major agronomic traits in soybean [22,23,24]. Previous studies using linkage mapping and genome-wide association studies (GWAS) have identified several QTLs and candidate genes associated with conventional pod-related traits, including pod size, pod color, pod number, and pod shattering, providing an important foundation for understanding the genetic basis of soybean pod-related traits [3,4,5,22,23,25,26,27,28,29,30]. For instance, in our previous study, mature pod images collected in 2017 and 2018 from the 284-line interspecific RIL population (NJRINP) were analyzed to obtain pod length and width from the minimum enclosing rectangle, projected area, the length-to-width ratio, and dominant-color values; these traits were subsequently used for linkage mapping [23]. Meanwhile, advances in image-based phenotyping have enabled the extraction of increasingly diverse morphological descriptors from pod images, substantially expanding the range of quantifiable pod phenotypes [3,10,16,20,21,23,24]. In our recent study, the DeepLabV3Binary workflow was applied to mature pod images from a cultivated soybean association panel of 187 accessions, and the resulting eight image-derived traits were evaluated through GWAS [24]. However, genetic analyses of such image-derived morphological descriptors remain relatively limited, and their genetic basis remains incompletely understood [3,21,23,24]. Integrating image-derived morphological descriptors with QTL mapping therefore provides an opportunity to further characterize genetic variation underlying soybean pod morphology and advance the genetic dissection of pod-related traits.

In the present study, we investigated the genetic basis of image-derived pod-related traits in an interspecific soybean RIL population. Eight quantitative pod-related traits were extracted from the reused NJRINP mature pod images using the established deep learning-based image phenotyping framework and subsequently subjected to composite interval mapping (CIM) based on a high-density genetic linkage map. Major QTL regions associated with these traits were identified, and candidate genes within these regions were further prioritized through genome annotation, functional information, and pod and seed developmental expression profiles. Our findings provide insights into the genetic architecture of soybean pod-related traits and offer valuable genetic resources for future soybean genetic improvement and molecular breeding.

## 2. Materials and Methods

### 2.1. Mature Soybean Pod Image Acquisition and Preprocessing

The mature soybean pod image dataset used in this study was derived from the interspecific recombinant inbred line (RIL) population (NJRINP), which was developed from a cross between the cultivated soybean cultivar NN86-4 and the wild accession PI 342618B. NN86-4 is characterized by tan, relatively large pods and a 100-seed weight of approximately 17.9 g, whereas PI 342618B exhibits black, small pods and a 100-seed weight of approximately 1.1 g [23]. Field trials were conducted at the Jiangpu Experimental Station of Nanjing Agricultural University (31°02′ N, 118°04′ E) in 2017 and 2018 using a completely randomized design with three replications. Each RIL was grown in a 2 m row with five hills per row. For each RIL in each year, five representative mature pods were randomly collected from each of the three field replications at physiological maturity. In both years, image acquisition followed the same standardized procedure described previously [23], and each sampled pod was photographed using the same imaging setup, including an industrial camera equipped with a 50 mm lens and mounted on a fixed stand, a white background, controllable illumination with backlight and LED lighting, and consistent pod positioning. White-balance correction was performed by setting the initial RGB values of the white background to 255, 255, and 255; however, no physical color reference chart was included. The final image resolution was 2048 × 1536 pixels. In the present study, the mature pod image dataset originally acquired and analyzed by Chang et al. [23] from the 2017 and 2018 NJRINP field trials was reused to extract and quantify pod-related traits using the image-analysis workflow described in Section 2.2.

Prior to trait extraction, all pod images were subjected to standardized preprocessing to ensure image quality and analytical consistency. The preprocessing procedure mainly included image quality inspection, background normalization, image cropping, and image format standardization, generating uniform RGB images suitable for subsequent image analysis and quantitative measurement of pod-related traits.

### 2.2. Pod Image Feature Extraction and Measurement of Pod-Related Traits

A deep learning-based image phenotyping framework reported in our recent study [24] was applied to extract quantitative descriptors from mature soybean pod images (Figure S1). The framework consisted of three major steps: (i) pod segmentation from RGB images; (ii) extraction of morphological and color descriptors from segmented pod regions; and (iii) calculation of quantitative traits representing pod shape, size, and color characteristics.

For pod segmentation, DeepLabV3Binary was implemented by adapting the DeepLabV3 semantic segmentation framework [31] with a ResNet50 backbone [32] for binary foreground segmentation. The DeepLabV3Binary model applied here was developed and evaluated in our recent study [24]. Model development used 188 pod images from four image datasets acquired using a common image-acquisition procedure, with each image obtained from a distinct genotype and individual plant. Pod regions were manually annotated with polygon labels using LabelMe, and the annotated images were randomly divided at the image level into training and validation sets at a ratio of 9:1. No independent test set was retained. The model was trained using the AdamW optimizer and a combined loss function (*L* = *L*_focal_ + 2*L*_dice_), and the model with the highest validation IoU was retained. Segmentation performance was evaluated on the validation set by comparing the predicted masks with the corresponding manually annotated masks. The best validation IoU, Dice coefficient, precision, and recall were 0.9774, 0.9885, 0.9880, and 0.9892, respectively [24]. The model generated binary pod masks by separating pod regions from the background for subsequent morphological and color trait extraction. During inference, predicted masks were restored to the original image dimensions, and connected-component analysis combined with morphological post-processing was performed to obtain continuous pod regions. Based on the segmented pod regions, skeleton-, contour-, and width-based measurements were used to characterize pod morphology. The extracted morphological traits included pod skeleton length (PSL), pod mean width (PMW), pod area (PA), pod elongation (PE = PSL/PMW), and pod mean curvature (PMC). Among these traits, PMW and PMC were calculated using the procedures implemented in the phenotyping pipeline reported in our recent study [24]. PMW was obtained by averaging widths sampled across the central 60% (from 20% to 80%) of the longitudinal span of the aligned binary pod mask and was expressed in cm. PMC, corresponding to pod curvature (PC) in that study, was obtained as the mean curvature of the smoothed main skeleton path and expressed in cm^−1^.

Color component traits were calculated from segmented pod regions after conversion from RGB to HSV color space. Hue (H) was expressed in degrees on a 0–360° scale, whereas saturation (S) and value (V) were expressed as normalized values ranging from 0 to 1. The mean H, S, and V of the pod pixels were used to quantify variation in pod color components. The complete image-analysis workflow, including pod segmentation, feature extraction, and trait calculation, was integrated into a graphical application named Soybean Analyzer, which enables automated extraction of pod-related traits from soybean pod images. Detailed definitions and calculation procedures for all extracted traits are provided in Supplementary Table S1. For each RIL year and field replication, the five sampled pods constituted biological subsamples, and their trait values were averaged to generate the plot-level phenotype used in subsequent statistical and QTL analyses.

### 2.3. Statistical Analysis

Descriptive statistics were performed to evaluate phenotypic variation among the RIL population. Variance components were estimated to assess the effects of genotype, year, and genotype-by-year interaction on pod-related traits. Broad-sense heritability (*H*^2^) on an across-year entry-mean basis was calculated according to Nyquist and Baker [33] as follows:

(1) $$H^{2}=\sigma_{g}^{2}/\left(\sigma_{g}^{2}+\frac{\sigma_{gy}^{2}}{n}+\frac{\sigma_{e}^{2}}{nr}\right)$$

Single-year *H*^2^ estimates were calculated as follows:

(2) $$H^{2}=\sigma_{g}^{2}/\left(\sigma_{g}^{2}+\sigma_{e}^{2}\right)$$

where $\sigma_{g}^{2}$ is the genotypic variance, $\sigma_{gy}^{2}$ is the genotype-by-year interaction variance, $\sigma_{e}^{2}$ is the residual error variance, *n* is the number of years (*n* = 2), and *r* is the number of replications within each year (*r* = 3). Because the single-year and across-year entry-mean *H*^2^ estimates were calculated on different bases, they were interpreted separately rather than compared directly.

Best linear unbiased predictions (BLUPs) for individual RILs were estimated using a linear mixed model, implemented with the R package *lme4* [34], with genotype, year, genotype-by-year interaction, and replication nested within year treated as random effects. The resulting BLUP estimates were used as integrated phenotypic values for subsequent QTL analysis. Pearson correlation analyses among pod-related traits were performed using phenotypic data obtained from 2017, 2018, and BLUP estimates. Potential outliers were checked against the corresponding original images and segmentation masks for possible errors; no observations were corrected or excluded. All statistical analyses were performed using R software (version 4.4.0) [35], and statistical significance was determined at *p* < 0.05.

### 2.4. Linkage Map and QTL Analysis

The previously developed high-density linkage map of the NJRINP population was used for QTL mapping [36]. Composite interval mapping (CIM) was performed using Windows QTL Cartographer 2.5 software with a 10-centimorgan (cM) window size, a 1-cM walk speed, and five control markers selected by the program’s stepwise regression procedure [37]. Phenotypic data from 2017 and 2018, together with the BLUP estimates, were used for QTL detection. Genome-wide LOD significance thresholds were determined by 1000 permutation tests [38], and the specific thresholds are provided in the note to Table 1. A QTL was declared when its LOD score exceeded the corresponding threshold, and its interval was defined by the 1-LOD support interval. The percentage of phenotypic variance explained (PVE) and additive effects were estimated for each detected QTL. QTLs detected across the 2017, 2018, and BLUP datasets were considered the same QTL when their 1-LOD support intervals overlapped. Because the BLUP estimates were derived from the 2017 and 2018 phenotypic data, only QTLs detected in both 2017 and 2018 were classified as cross-year stable; those detected in one year and the BLUP dataset were classified as BLUP-supported. The nomenclature of QTLs identified in this study followed the principles described by McCouch et al. [39].

### 2.5. Candidate-Gene Prioritization

Candidate gene analysis focused primarily on major genomic regions containing cross-year stable QTLs. The *qPE11* signal, detected in 2017 and in the BLUP dataset, was also retained for exploratory candidate gene analysis as a BLUP-supported QTL. The genetic positions of the selected QTLs were converted to physical coordinates based on the soybean Williams 82 (Wm82.a1) reference genome, and annotated genes within the corresponding intervals were retrieved from SoyBase (https://www.soybase.org/) [40]. Wm82.a1 coordinates were retained to preserve direct comparability with previous analyses of the same NJRINP population and linkage map [23,36]. Gene-model correspondences in Wm82.a4 were obtained using the SoyBase Gene Model Translation/Correspondence tool. Genes within selected focal QTL intervals were prioritized based on their genomic positions, functional annotations from SoyBase, previously reported soybean pod- or seed-related QTLs and genes, functional information from *Arabidopsis* orthologs, and public soybean pod and seed developmental expression profiles based on the Wm82.a1 reference genome. Public expression profiles were used only as supporting evidence for candidate-gene prioritization.

## 3. Results

### 3.1. Descriptive Statistics and Heritability of Pod-Related Traits in the NJRINP

**Figure 1:**
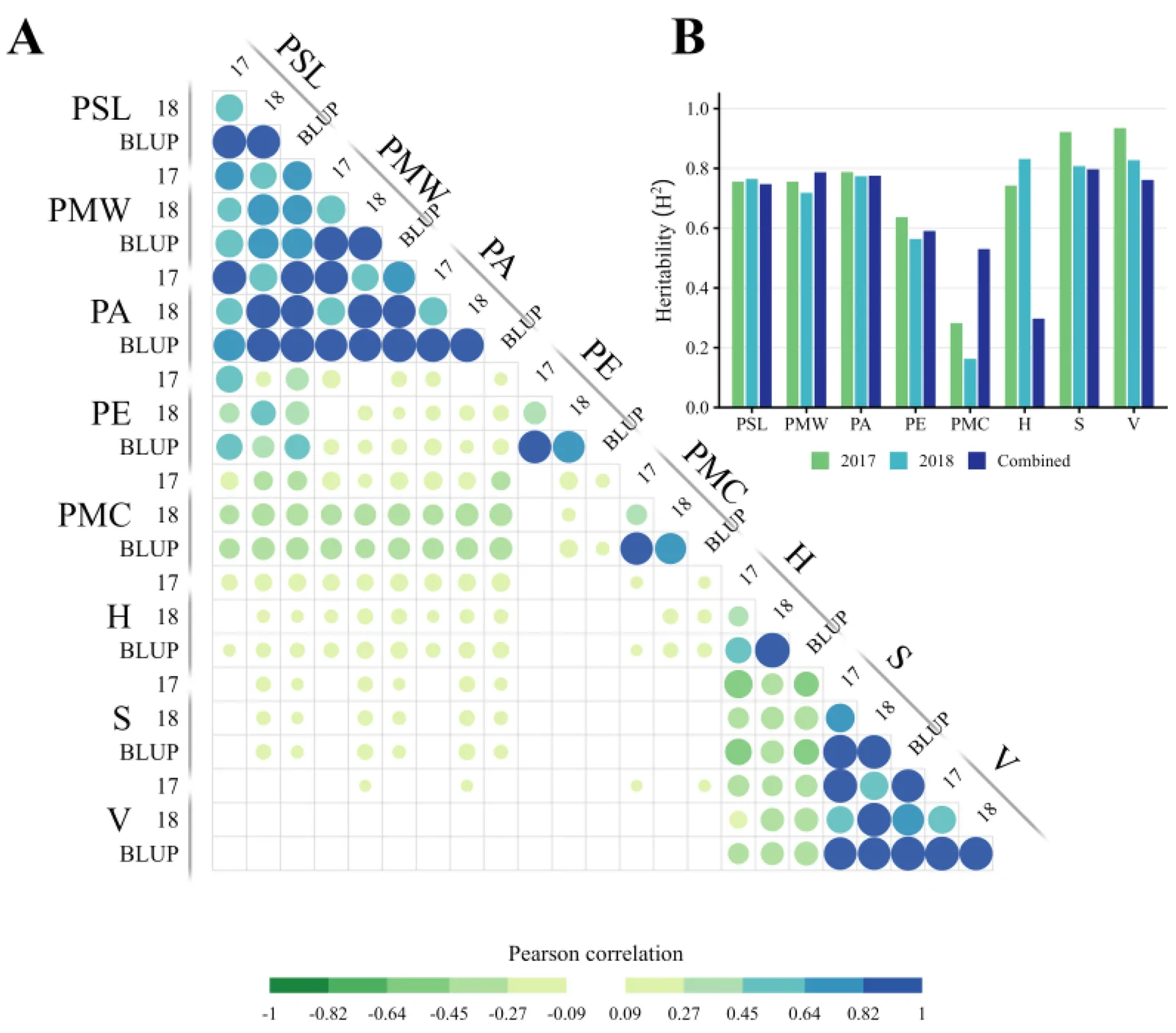
Pearson correlations and broad-sense heritability of eight image-derived pod-related traits in the NJRINP population: (**A**) Pearson correlation matrix among the 2017 (17), 2018 (18), and BLUP values of PSL, PMW, PA, PE, PMC, H, S, and V, including cross-year correlations. The same ordered variables are represented on both axes; only the lower triangle is shown, and diagonal self-correlations (*r* = 1) are omitted. Accordingly, PSL-17 and V-BLUP are displayed only on the diagonal and vertical axes, respectively. Circle color indicates the direction of correlation, with green and blue representing negative and positive correlations, respectively, whereas circle size indicates the absolute magnitude of the correlation coefficient. (**B**) Single-year H^2^ estimates for 2017 and 2018 and across-year entry-mean H^2^ estimates from the combined analysis of both years. The two types of estimates were calculated using Equations (2) and (1), respectively, and should not be compared directly. PSL, pod skeleton length; PMW, pod mean width; PA, pod area; PE, pod elongation; PMC, pod mean curvature; H, hue; S, saturation; V, value; BLUP, best linear unbiased prediction.

A wide range of phenotypic variation was observed among the 284 RILs of the NJRINP population for eight image-derived pod-related traits evaluated in two years (2017 and 2018), including five morphological traits (PSL, PMW, PA, PE, and PMC) and three color component traits (H, S, and V) (Table S2; Figure S2). The traits exhibited continuous variation, although their distributional shapes differed among traits and years. PE and several color traits deviated from approximate normality, with S and V showing multimodal distributions (Figure S2). H also showed a marked between-year shift, with RIL means of 44.50° in 2017 and 64.12° in 2018. The coefficients of variation (CVs) ranged from 7.69% to 30.46% across traits and years. Among the morphological traits, PA exhibited the greatest variation (CV values of 20.32% and 21.80% in 2017 and 2018, respectively). The color component traits also varied considerably, with CV values reaching 26.32% for S in 2018 and 30.46% for V in 2017. Single-year H^2^ estimates ranged from 0.16 to 0.93, whereas across-year entry-mean H^2^ estimates ranged from 0.30 to 0.80 (Table S2; Figure 1B). PMC showed low single-year H^2^ estimates of 0.28 in 2017 and 0.16 in 2018, whereas its across-year entry-mean H^2^ estimate was 0.53. The across-year entry-mean H^2^ estimates were 0.59 for PE; 0.75, 0.79, and 0.78 for PSL, PMW, and PA, respectively; 0.30 for H; and 0.80 and 0.76 for S and V, respectively. Overall, the moderate-to-high heritability observed for most pod-related traits indicated that the image-derived phenotypic descriptors captured substantial genetic variation within the NJRINP population.

Pearson correlation analyses revealed consistent relationships among pod-related traits across the 2017, 2018, and BLUP datasets (Figure 1A). Pod shape-related traits displayed weaker correlations with pod size-related traits, indicating that these traits captured partially independent aspects of pod morphology. In contrast, strong positive correlations were observed among pod size-related traits, particularly between PA and PSL (*r* = 0.90–0.94) and between PA and PMW (*r* = 0.85–0.92), suggesting coordinated variation among pod size-related characteristics. PSL and PMW also showed significant positive correlations across datasets. Pod color component traits displayed distinct correlation patterns, with strong positive correlations between S and V (*r* = 0.80–0.93), while H showed negative correlations with S and V in the BLUP dataset. The consistency of major correlation patterns across years and BLUP estimates supported the reliability of the extracted pod-related traits for subsequent QTL mapping.

### 3.2. Linkage Mapping of Pod-Related Traits in the NJRINP

**Figure 2:**
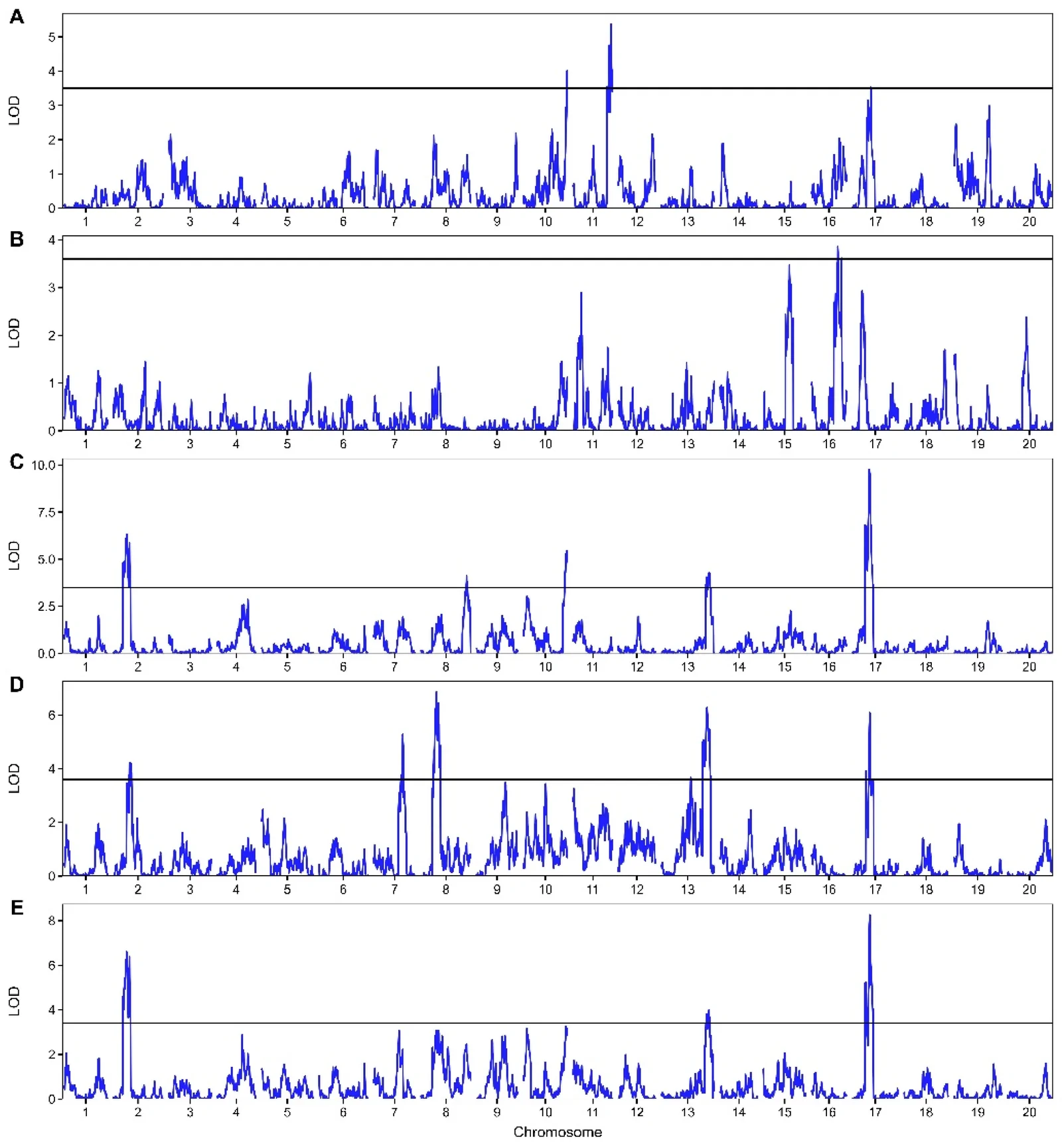
Genome-wide LOD profiles of five pod morphological traits based on BLUP estimates in the NJRINP population: (**A**) PE, (**B**) PMC, (**C**) PSL, (**D**) PMW, and (**E**) PA. The x-axis indicates the 20 soybean chromosomes, and the y-axis indicates the LOD score. Black horizontal lines indicate the trait-specific LOD significance thresholds determined by 1000 permutation tests. QTLs detected in individual years (2017 and 2018) are summarized in Table 1.

**Figure 3:**
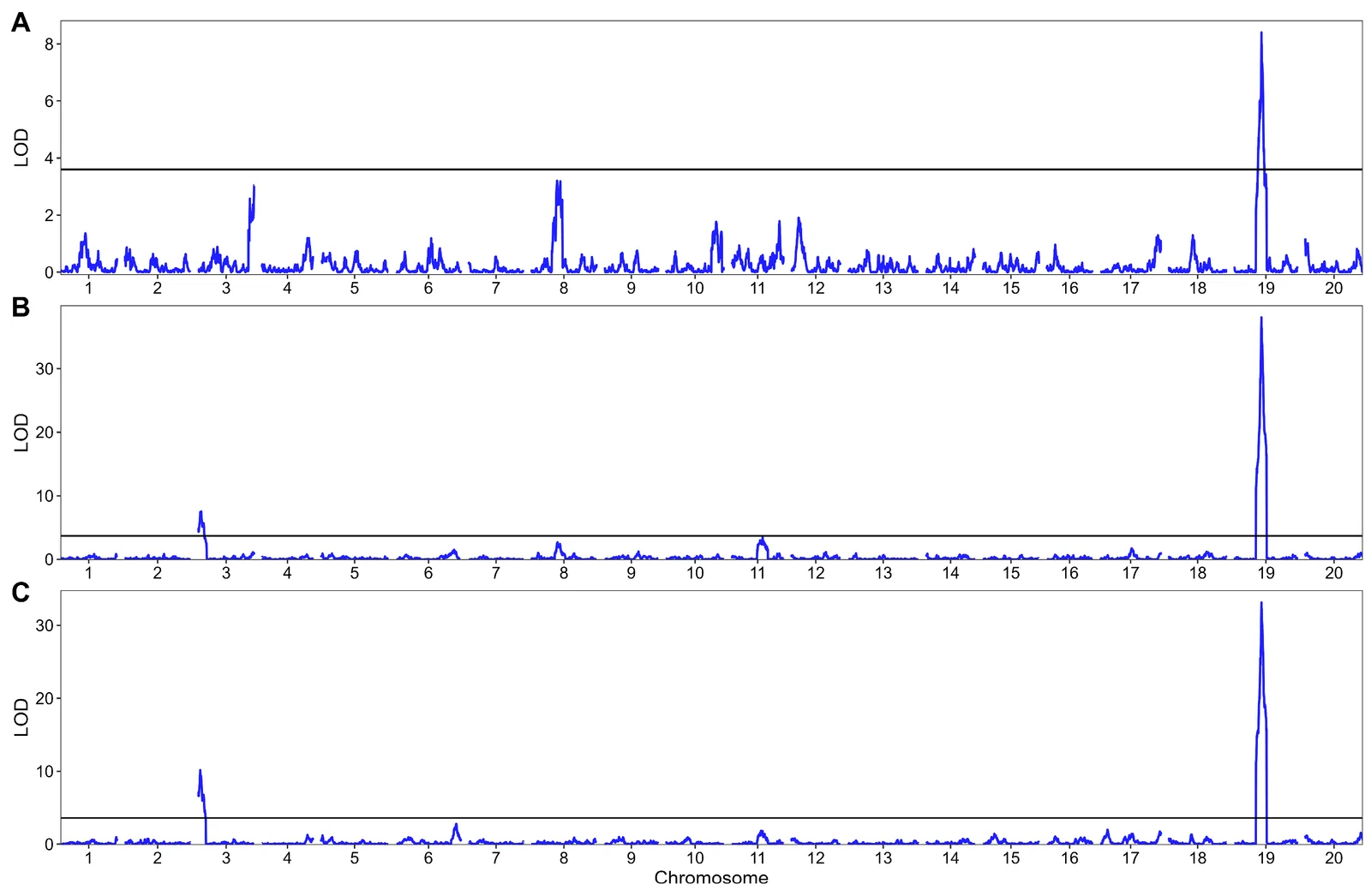
Genome-wide LOD profiles of three pod color component traits based on BLUP estimates in the NJRINP population: (**A**) H, (**B**) S, and (**C**) V. Black horizontal lines indicate the trait-specific LOD significance thresholds determined by 1000 permutation tests. QTLs detected in individual years (2017 and 2018) are summarized in Table 1.

A total of 54 QTLs were detected for the image-derived pod-related traits across the 2017, 2018, and BLUP datasets (Table 1). Among these, 17, 13, and 24 QTLs were identified in the 2017, 2018, and BLUP datasets, respectively. BLUP-based LOD profiles are presented in Figure 2 and Figure 3. Based on the overlap of 1-LOD support intervals, these QTLs were integrated into 39 non-redundant QTLs distributed across 11 soybean chromosomes. These QTLs covered diverse categories of pod-related traits, including pod shape, size, and color components. Specifically, 9 QTLs were identified for PSL, 9 for PMW, 7 for PA, 6 for PE, 1 for PMC, 2 for H, 3 for S, and 2 for V. The detected QTLs exhibited a wide range of statistical support and phenotypic contributions, with LOD values ranging from 3.55 to 44.64 and PVE values ranging from 3.47% to 50.31% (Table 1). Most QTLs exhibited minor or moderate effects, whereas several explained more than 10% of phenotypic variation. Among morphological traits, relatively large-effect QTLs were mainly detected for pod size-related traits, particularly those located on chromosome 17. Pod color component traits, especially S and V, also showed strong phenotypic effects. Notably, the estimated PVE values of *qS19-1* and *qV19* were 50.31% and 43.73%, respectively. These large estimates should be regarded as population- and model-specific and interpreted cautiously in view of the multimodal distributions of S and V. These results demonstrated that the recalculated image-derived traits captured substantial genetic variation suitable for genetic dissection. The correspondence of these selected QTL regions with those reported in our previous NJRINP study [23] is summarized in Table S8.

To identify robust genetic loci associated with pod-related traits, QTLs repeatedly detected across different datasets were further evaluated. Six QTLs were consistently detected in both 2017 and 2018 and were classified as cross-year stable QTLs, including *qPSL17-1*, *qPA17*, *qS3*, *qV3*, *qS19-1*, and *qV19*. In addition, *qPMW17-1*, *qPE11*, and *qH19* were detected in 2017 and in the BLUP dataset and were classified as BLUP-supported QTLs. On chromosome 17, the intervals of the cross-year stable *qPSL17-1* and *qPA17* overlapped with that of the BLUP-supported *qPMW17-1*, defining a pod size-related QTL region. For pod color components, the cross-year stable *qS3* and *qV3* showed overlapping intervals on chromosome 3. On chromosome 19, the intervals of the cross-year stable *qS19-1* and *qV19* partially overlapped, and the BLUP-supported *qH19* interval overlapped both the *qS19-1* and *qV19* intervals. These focal QTL regions were subsequently selected for candidate-gene prioritization.

### 3.3. Prioritization of Candidate Genes for Major QTLs Underlying Pod-Related Traits

Based on the cross-year stable and BLUP-supported QTLs identified above, candidate genes within major QTL regions were further prioritized by integrating genomic annotation, functional information, and developmental expression profiles from soybean pod and seed tissues. The selected QTLs were grouped into four major genomic regions associated with pod size, pod shape, and pod color component traits.

The *qPSL17-1*, *qPMW17-1*, and *qPA17* loci on chromosome 17 represented the most prominent genomic region associated with pod size-related variation. *qPSL17-1* and *qPA17* were cross-year stable, whereas *qPMW17-1* was BLUP-supported. These three QTLs showed overlapping physical intervals containing *Glyma.17G109100* (*GmSW17*; formerly *Glyma17g11760*). Because *GmSW17* is a validated regulator of soybean seed size [41], it was retained as a positional candidate for pod size-related variation; however, its role in mature pod-wall development requires direct validation. Moreover, several genes within this interval, including *Glyma17g11940* and *Glyma17g11790*, exhibited comparatively high expression levels in public pod-development datasets (Table S4; Figure S3); these expression patterns were used only as exploratory supporting information. For pod shape-related traits, the BLUP-supported *qPE11* interval on chromosome 11 contained several genes with potential developmental functions (Table S5; Figure S4). Among them, *Glyma11g33560*, *Glyma11g33520*, and *Glyma11g34630* were retained as exploratory positional candidates based on their functional annotations and comparatively high expression in public pod and seed datasets; their roles in pod elongation require further validation.

For pod color component traits, cross-year stable loci on chromosomes 3 and 19 provided further evidence for the genetic basis of soybean pod pigmentation (Table S3). On chromosome 19, *qS19-1* and *qV19* had partially overlapping intervals, and the BLUP-supported *qH19* interval overlapped both. *Glyma.19G120400* (*L1*; formerly *Glyma19g29880*) was located within the *qV19* interval but outside the *qS19-1* and *qH19* intervals and the shared interval of *qS19-1* and *qV19*. *L1* has previously been validated as the causal gene underlying soybean pod color variation, and the presence of this gene within the *qV19* interval provides independent biological support for the accuracy of *qV19* localization (Table S6). In addition, the overlapping *qS3* and *qV3* intervals on chromosome 3 contained *Glyma.03G005700* (formerly *Glyma03g00800*), which encodes an HMGL-like protein with a conserved LeuA allosteric domain similar to *L1* (Table S7). The homologous relationship between these two genomic regions [42], together with their developmental expression profiles (Figures S5 and S6), suggests that *Glyma03g00800* may represent a positional candidate gene contributing to soybean pod color variation, although further validation is required.

Collectively, the integration of QTL position, functional annotation, and pod and seed developmental expression profiles prioritized several genes within the selected QTL regions. Among them, *L1*, a previously validated causal gene underlying soybean pod color variation, was located within *qV19*, whereas *GmSW17*, a functionally characterized regulator of soybean seed size [41], was prioritized as a positional candidate for the pod size-related QTL region located on chromosome 17.

## 4. Discussion

### 4.1. Deep Learning-Assisted Image Phenotyping Expands the Phenotypic Characterization of Soybean Pod-Related Traits

Accurate phenotypic characterization is fundamental for dissecting the genetic basis of complex agronomic traits. In soybean, pod-related traits have traditionally been evaluated through manual measurements and visual assessments, with parameters such as pod length, width, and general appearance serving as the primary descriptors for genetic analysis and breeding applications [2,3]. Although these conventional approaches have contributed substantially to soybean genetic improvement, they mainly capture qualitative appearance or overall size and provide limited information on complex morphological variation. In particular, subtle variations in pod shape, curvature, and color components are difficult to quantify accurately using conventional approaches, potentially resulting in the underestimation of phenotypic diversity associated with pod growth and development. Therefore, expanding the dimensionality and precision of pod phenotyping is essential for uncovering the genetic basis of soybean pod variation.

Recent advances in high-throughput phenotyping and computer vision technologies have provided new opportunities to overcome these limitations by enabling objective and quantitative characterization of complex plant traits [12,14,16]. Deep learning-based image analysis has further enhanced the accuracy and robustness of image segmentation and trait extraction and has been increasingly applied to phenotyping studies in soybean and other crops [9,10,16,17,18,19,20,21]. However, most previous studies have mainly focused on improving phenotyping efficiency or developing automated measurement platforms, whereas the integration of image-derived descriptors into genetic analyses remains relatively limited [8,9,10,20,21]. Our recent study applied the DeepLabV3Binary workflow to mature-pod images from a cultivated soybean association panel of 187 accessions and evaluated the resulting eight image-derived traits through GWAS [24]. In the present study, the same workflow and trait definitions were applied to the reused images of the 284-line interspecific NJRINP population [23] to calculate eight quantitative traits representing pod size (PSL, PMW, and PA), shape characteristics (PE and PMC), and color components (H, S, and V); these traits were subsequently evaluated through linkage mapping rather than GWAS. These image-derived traits exhibited substantial phenotypic variation (CV = 7.69–30.46%) and across-year entry-mean *H*^2^ estimates ranging from 0.30 to 0.80 in the NJRINP population, demonstrating that they captured genetically meaningful variation rather than merely visual differences. Moreover, the distinct correlation patterns among size-, shape-, and color-related traits indicated that these descriptors represented both coordinated and partially independent aspects of soybean pod morphology.

Relative to the image-derived measurements previously analyzed in the NJRINP population [23], PSL and PMW provide alternative descriptions of pod length and width based on skeleton length and central-region mean width, respectively, while PMC quantifies pod curvature. PE is conceptually equivalent to the previously reported pod length-to-width ratio (PRLW), although PE was calculated from PSL and PMW rather than from the minimum-enclosing-rectangle-derived PL and PW used previously [23]. PA represents projected pod area in both analyses, whereas the current mean HSV values and the previously reported dominant-color values both characterize pod color but use different pixel-level summaries. Therefore, the present study should be regarded as an extension of the previous analysis through the application of alternative image-derived descriptors and their subsequent genetic evaluation, rather than as the generation of a new image dataset or segmentation model.

### 4.2. Genetic Dissection of Pod-Related Traits Derived from Image-Based Phenotyping Through QTL Mapping

The identification of 54 QTLs, subsequently integrated into 39 non-redundant loci across 11 chromosomes, demonstrates that the image-derived pod-related traits captured extensive and genetically controlled variation in the NJRINP population. Most loci explained small-to-moderate proportions of phenotypic variation, consistent with the quantitative inheritance of soybean pod-related traits reported previously [22,23]. Notably, more QTLs were detected using BLUP values than in either individual year, likely reflecting improved mapping precision through the integration of phenotypic information across years and the reduction in year-specific environmental effects.

Six QTLs, namely *qPSL17-1*, *qPA17*, *qS3*, *qV3*, *qS19-1*, and *qV19*, were detected in both 2017 and 2018 and were therefore classified as cross-year stable. In contrast, *qPMW17-1*, *qPE11*, and *qH19* were detected in one year and in the BLUP dataset and were classified as BLUP-supported. Among these loci, the chromosome 17 pod size-related region was particularly notable because *qPSL17-1*, *qPMW17-1*, and *qPA17* formed a QTL cluster with overlapping 1-LOD intervals. The co-localization of these QTLs was consistent with the strong correlations among PSL, PMW, and PA, suggesting that this region contributes to the coordinated regulation of multiple pod size dimensions. However, the current linkage analysis cannot distinguish whether these effects result from a single pleiotropic locus or multiple closely linked genes, and further fine mapping and recombinant analysis will be required to resolve the underlying genetic mechanism. In addition, the BLUP-supported *qPE11* demonstrates that proportional pod geometry may have a distinct genetic basis from overall pod size, although this signal requires confirmation in independent datasets. Similarly, the detection of QTLs for PMC suggests that image-derived shape descriptors can reveal genetic variation that may be difficult to capture using conventional measurements. Nevertheless, the relatively lower stability of several shape-related QTLs across years indicates that these traits may be more sensitive to developmental or year-specific environmental variation than pod size-related traits.

For pod color component traits, the cross-year stable QTLs *qS3* and *qV3* on chromosome 3 and *qS19-1* and *qV19* on chromosome 19 were identified. The BLUP-supported *qH19* interval also overlapped the chromosome 19 color-related QTL region but was not considered cross-year stable. Although the imaging setup, illumination configuration, and white-balance procedure were standardized across both years, the absence of a physical color reference chart should be considered when interpreting the observed between-year differences in H, S, and V. The BLUP-specific negative correlations of H with S and V were not observed in either individual year, indicating that these relationships were not stable across years. These inconsistencies, together with the marked difference in H between years and its relatively low across-year entry-mean heritability, indicate that the BLUP-supported *qH19* signal should be interpreted with caution. The overlapping *qS3* and *qV3* intervals and the partially overlapping *qS19-1* and *qV19* intervals revealed genomic regions associated with multiple quantitative color components. These QTLs correspond to previously reported pod color QTLs detected using image-based pod color measurements in soybean [23]. The chromosome 3 region is associated with the classical pod-color locus *L2*, whereas *qV19* encompasses *L1* [4,26,43]. The large effects of *qS19-1* and *qV19* indicate that quantitative measurements of saturation and brightness can effectively capture major pigmentation loci traditionally identified through categorical pod-color phenotypes. Moreover, the strong correlation between S and V suggests that the overlap between their QTL intervals may reflect the influence of a common pigmentation locus on multiple pod color components.

Comparison of genetic and physical intervals with those reported in the previous NJRINP study [23] showed that the focal loci on chromosomes 3, 17, and 19 corresponded to or were located near previously reported QTL regions, whereas no corresponding PRLW QTL on chromosome 11 was reported previously (Table S8). Thus, the BLUP-supported *qPE11* represents the principal additional focal signal identified here. Collectively, these results provide a complementary genetic characterization of soybean pod-related traits using alternative image-derived descriptors, particularly for the pod size-related cluster on chromosome 17 and the pod color-related loci on chromosomes 3 and 19. However, because linkage-mapped QTL intervals may contain multiple genes, further validation through higher-resolution mapping and functional analyses will be required before these loci can be reliably utilized in breeding programs.

### 4.3. Candidate Genes Underlying Major QTLs Provide Insights into the Genetic Basis of Soybean Pod-Related Traits

Identifying functional genes underlying QTLs remains challenging because linkage intervals generally contain multiple genes with diverse biological functions. In this study, candidate genes within the selected QTL regions were prioritized by integrating genomic annotation, previous functional studies, and pod and seed developmental expression profiles. This integrated strategy provided biological interpretation of the selected QTL regions identified through image-derived phenotyping and facilitated candidate gene prioritization.

The chromosome 17 pod size-related QTL cluster harboring *qPSL17-1*, *qPMW17-1*, and *qPA17* represented a focal region for candidate-gene prioritization. *GmSW17* was located within the overlapping intervals of these three QTLs and encodes a ubiquitin-specific protease involved in a deubiquitinase module with GmSGF11 and GmENY2. Previous studies demonstrated that natural variation in *GmSW17* affects histone H2B ubiquitination, the expression of the cell cycle regulator GmDP-E2F-1, and downstream cell division and expansion processes, ultimately influencing soybean seed width and weight [41]. The location of *GmSW17* within these overlapping QTL intervals supports its prioritization as a positional candidate for pod size-related variation. However, its direct role in mature pod-wall development remains to be experimentally demonstrated. The available expression atlas includes pod-shell samples only at 10 and 14 DAF, and expression at these stages does not establish such a role. A parent-specific *GmSW17* haplotype comparison between NN86-4 and PI 342618B was not performed and should be included in subsequent validation.

*L1*, located within *qV19* but outside *qS19-1, qH19,* and the overlapping interval of *qS19-1* and *qV19,* has previously been validated as the causal gene underlying soybean pod color variation. *L1* encodes a hydroxymethylglutaryl-coenzyme A (CoA) lyase-like (HMGL-like) protein functioning as a eucomic acid synthase and promotes the accumulation of eucomic and piscidic acids, thereby affecting pod and seed-coat pigmentation and contributing to pod shattering regulation [4]. The recovery of *L1* within the cross-year stable *qV19* further demonstrates that this QTL represents a quantitative re-detection of the previously characterized *L1* locus. The partially overlapping *qS19-1* signal is interpreted as an associated saturation QTL, but its interval does not contain *L1*. In addition, *Glyma.03G005700*, located within the overlapping *qS3* and *qV3* intervals, shares a conserved HMGL-like/LeuA domain with *L1*. Because parent-specific polymorphisms and eucomic and piscidic acid contents were not examined, this domain similarity is treated only as exploratory positional evidence, and no causal role is inferred. The genes highlighted within the BLUP-supported *qPE11* interval should likewise be regarded as exploratory positional candidates. Further functional validation through fine mapping, parental polymorphism analysis, metabolite measurement, and functional assays will be required to confirm their contributions to soybean pod-related trait variation.

## 5. Conclusions

This study applied an established deep learning-based image phenotyping framework to characterize and genetically dissect soybean pod-related traits in an interspecific soybean RIL population. Eight image-derived traits representing pod size, shape, and color components exhibited substantial phenotypic variation and genetic control, providing additional dimensions beyond conventional pod measurements. QTL mapping identified six cross-year stable and three BLUP-supported QTLs, most of which corresponded to previously implicated regions. Candidate-gene analysis prioritized *GmSW17* and other genes as positional candidates requiring further validation, whereas *L1* is a previously validated causal gene located within *qV19*. These findings demonstrate the effectiveness of combining deep learning-based phenotyping with genetic analysis for dissecting the genetic architecture of complex soybean pod-related traits and provide valuable phenotypic resources, stable QTLs, and candidate genes for future functional studies and molecular breeding.

## Figures and Tables

**Table 1:** Summary of QTL mapping results for image-derived pod-related traits in the NJRINP population.

Trait	QTL	Gm	LOD	CI (cM)	PI (bp)	AE ^a^	PVE (%)	Year	Known QTLs/Genes ^b^
PSL	*qPSL2-1*	2	6.11	29.00–32.30	5,844,866–6,281,070	0.12	7.48	2017	*Seedheight2-1*
	*qPSL2-2*	2	4.69	23.70–25.80	4,859,713–5,238,951	0.10	5.49	2018	*Seedheight2-1*
	*qPSL2-3*	2	6.31	32.90–35.40	6,397,100–6,888,682	0.07	6.62	BLUP	*Seedheight2-1*
	*qPSL8*	8	4.12	110.60–115.80	41,297,993–42,366,646	0.06	4.30	BLUP	*Seedweight34-13*;*Seedweight36-1*
	*qPSL9*	9	3.55	38.80–41.00	6,060,636–7,197,218	0.09	4.35	2017	*Seedweight35-6*
	*qPSL10*	10	5.44	108.00–110.30	48,150,130–48,554,070	0.07	5.68	BLUP	*Seedweightperplant3-2*
	*qPSL13*	13	4.05	113.50–116.50	38,785,190–39,219,819	0.06	4.23	BLUP	*Seedweight45-6*
	***qPSL17-1***	17	9.67	42.10–45.00	8,680,924–9,321,459	0.15	12.56	2017	***GmSW17(Glyma17g11760)***
			6.71	41.80–45.50		0.12	8.12	2018	
			9.77	42.80–45.10		0.09	10.78	BLUP	
	*qPSL17-2*	17	6.82	32.40–33.50	6,647,575–7,070,746	0.08	7.70	BLUP	*Seedweight50-1*;*Seedweight50-11*
PMW	*qPMW2*	2	4.21	43.40–46.70	8,010,293–9,073,201	0.01	4.85	BLUP	Noneidentified
	*qPMW7*	7	5.29	72.30–74.00	18,260,291–19,127,677	0.01	6.16	BLUP	*Seedweight12-4*
	*qPMW8-1*	8	5.27	48.10–49.10	10,649,165–10,978,688	0.02	6.65	2017	*Seedweight35-1*
	*qPMW8-2*	8	6.86	37.80–38.80	7,719,214–7,846,413	0.01	8.06	BLUP	*Seedlength5-1*
	*qPMW8-3*	8	6.45	42.30–43.00	8,856,488–9,306,257	0.01	7.60	BLUP	*Seedweight30-4*;*Seedweight6-2*
	*qPMW10*	10	4.36	13.30–17.50	2,703,497–3,480,831	0.02	5.83	2018	Noneidentified
	*qPMW13*	13	6.28	112.40–118.10	38,576,022–39,635,577	0.01	7.60	BLUP	*Seedweight45-6*
	*qPMW17-1*	17	6.13	42.50–45.20	8,739,495–9,251,289	0.02	7.84	2017	*GmSW17(Glyma17g11760)*
			6.09	43.10–45.10		0.01	7.14	BLUP	
	*qPMW17-2*	17	3.92	33.50–35.90	7,027,221–7,664,397	0.01	4.68	BLUP	*Seedweight18-2*
PA	*qPA2-1*	2	5.64	30.40–32.90	6,023,059–6,418,439	0.10	6.94	2017	*Seedheight2-1*
	*qPA2-2*	2	4.29	28.90–30.10	5,844,866–6,094,921	0.09	5.36	2018	*Seedheight2-1*
	*qPA2-3*	2	4.18	23.70–24.10	4,859,713–5,107,482	0.06	4.90	BLUP	*Seedheight2-1*
	*qPA2-4*	2	6.38	40.20–41.30	7,451,301–7,537,806	0.07	7.34	BLUP	Noneidentified
	*qPA8*	8	3.71	50.50–54.90	11,324,549–12,311,439	0.08	4.24	2017	*Seedweight35-1*
	*qPA9*	9	4.57	38.80–40.30	6,060,636–7,076,546	0.09	5.65	2017	*Seedweight35-6*
	***qPA17***	17	9.90	42.70–45.20	8,680,924–9,321,459	0.14	12.78	2017	*GmSW17(Glyma17g11760)*
			6.40	41.80–44.80		0.12	8.68	2018	
			8.26	43.00–45.30		0.08	9.61	BLUP	
PE	*qPE10*	10	4.01	109.50–110.30	48,444,400–48,554,070	0.05	4.78	BLUP	*Seedweightperplant3-2*
	*qPE11*	11	4.53	94.20–97.00	35,187,528–36,625,526	−0.13	6.28	2017	Noneidentified
			5.37	94.90–96.30		−0.05	6.53	BLUP	
	*qPE13*	13	3.73	78.00–79.10	30,902,703–31,177,845	−0.09	4.64	2018	*Seedweight49-13*;*Seedweight41-2*
	*qPE16*	16	3.75	20.90–25.30	3,619,974–4,347,585	−0.12	5.26	2017	*Seedweight2-6*;*Seedweight4-4*
	*qPE17*	17	5.12	22.70–25.60	4,689,974–5,293,446	0.10	6.37	2018	*Seedweight21-2*;*Seedweight22-3*
	*qPE19*	19	7.25	86.40–88.30	42,561,556–43,183,016	0.13	9.09	2018	*Seedlength1-10*;*cqSeedweight-005*
PMC	*qPMC16*	16	3.86	63.40–66.00	28,991,462–29,753,693	0.02	5.12	BLUP	Noneidentified
H	*qH3*	3	6.45	106.00–106.60	47,043,213–47,443,915	0.86	7.03	2017	
	*qH19*	19	15.30	52.00–56.20	36,542,873–37,594,044	−1.33	18.17	2017	
			8.40	51.20–52.50		−0.58	11.09	BLUP	
S	***qS3***	3	4.78	2.90–5.90	357,751–705,287	0.02	3.47	2017	
			5.52	4.40–6.30		0.02	6.31	2018	
			7.52	4.40–6.30		0.02	5.96	BLUP	
S	***qS19-1***	19	44.64	51.20–51.80	36,507,791–36,745,471	0.08	50.31	2017	
			17.83	51.10–51.80		0.05	23.19	2018	
			38.05	51.20–51.90		0.05	41.31	BLUP	
	*qS19-2*	19	8.66	42.10–42.90	34,502,613–34,654,302	0.03	12.16	2018	Noneidentified
V	***qV3***	3	9.93	3.30–6.40	411,496–705,287	0.04	8.14	2017	
			5.00	3.60–7.00		0.03	5.98	2018	
			10.17	3.20–4.40		0.03	8.80	BLUP	
	***qV19***	19	38.75	51.20–54.60	36,542,873–37,683,143	0.10	43.73	2017	*L1(Glyma19g29880)*
			14.05	51.80–57.40		0.06	18.36	2018	
			33.14	51.20–51.90		0.06	36.45	BLUP	

Note: QTL, quantitative trait locus; Gm, chromosome; LOD, logarithm of odds; CI, confidence interval; PI, physical interval based on Wm82.a1; AE, additive effect; PVE, phenotypic variance explained; BLUP, best linear unbiased prediction; PSL, pod skeleton length (cm); PMW, pod mean width (cm); PA, pod area (cm^2^); PE, pod elongation (dimensionless); PMC, pod mean curvature (cm^−1^); H, hue (0–360°); S, saturation (0–1); V, value (0–1); bp, base pair; cM, centimorgan. ^a^ Positive additive effect (AE) values indicate that the trait-increasing allele was contributed by NN86-4, whereas negative AE values indicate that it was contributed by PI 342618B. AE is expressed in the corresponding unit or scale of each trait. ^b^ Based on the QTL list on SoyBase (www.soybase.org) and the genes previously reported. “None identified” indicates that no overlapping previously reported gene or QTL was identified among the sources examined. The corresponding Wm82.a4 identifiers are *Glyma.17G109100* (*GmSW17*; formerly *Glyma17g11760*) and *Glyma.19G120400* (*L1*; formerly *Glyma19g29880*). Permutation-derived LOD thresholds for 2017, 2018, and BLUP, respectively: PSL = 3.5/3.5/3.5; PMW = 3.6/3.6/3.6; PA = 3.6/3.6/3.4; PE = 3.3/3.6/3.5; PMC = 3.6/3.6/3.6; H = 3.6/3.6/3.6; S = 3.8/3.6/3.7; and V = 3.6/3.6/3.6. Bold italic QTL names indicate cross-year stable QTLs; underlined italic QTL names indicate BLUP-supported QTLs.

## Data Availability

The data presented in this study are available within the article and Supplementary Materials. The Soybean Analyzer application is available from the corresponding author upon reasonable request; the trained DeepLabV3Binary model weights are not available for separate distribution.

## Supplementary Materials

The following supporting information can be downloaded at https://www.mdpi.com/article/10.3390/plants15182769/s1, Table S1: Definitions and interpretations of image-derived pod-related traits; Table S2: Descriptive statistics and heritability estimation of image-derived traits; Table S3: Summary of cross-year stable and BLUP-supported QTL regions associated with soybean pod-related traits and the number of annotated genes within the corresponding intervals; Table S4: Annotated genes within the focal QTL interval on chromosome 17 associated with soybean pod size-related traits; Table S5: Annotated genes within the BLUP-supported *qPE11* interval on chromosome 11 associated with soybean pod shape-related variation; Table S6: Annotated genes within the combined span of the *qS19-1*, *qV19*, and *qH19* intervals on chromosome 19 associated with soybean pod color component variation; Table S7: Annotated genes within the combined span of the *qS3* and *qV3* intervals on chromosome 3 associated with soybean pod color component variation; Table S8: Comparison of focal QTLs identified in the present study with corresponding QTLs reported by Chang et al. (2021) [23] in the NJRINP population; Figure S1: Deep learning-based workflow used for soybean pod image segmentation and quantitative trait extraction; Figure S2: Phenotypic distributions of eight image-derived pod-related traits; Figure S3: Developmental expression profiles of genes within the chromosome 17 pod size-related QTL region; Figure S4: Developmental expression profiles of genes within the BLUP-supported *qPE11* interval associated with pod elongation; Figure S5: Developmental expression profiles of genes within the cross-year stable *qS3* and *qV3* intervals on chromosome 3; Figure S6: Developmental expression profiles of genes within the combined span of the *qS19-1*, *qV19*, and *qH19* intervals on chromosome 19.
