# Supplementary material for: Genetic Dissection of Image-Derived Pod-Related Traits in an Interspecific Soybean RIL Population
Source: Plants (Basel). 2026 Sep 10;15(18):2769. doi: 10.3390/plants15182769 (PMC13609990; doi:10.3390/plants15182769)
Supplement: Supplementary file 1 [file plants-15-02769-s001.zip › Supplementary materials.pdf]

Genetic Dissection of Image-Derived Pod-Related Traits in an Interspecific Soybean RIL Population

Fangguo Chang[1,*], Tuanjie Zhao[2], Shunchang Su[3], Xiaohan Ruan[3], Liping Wei[3,*]

[1]College of Agronomy, Gansu Agricultural University, Lanzhou 730070, China

[2]Key Laboratory of Biology and Genetics Improvement of Soybean, Ministry of Agriculture; Zhongshan Biological Breeding Laboratory (ZSBBL); National Innovation Platform for Soybean Breeding and Industry-Education Integration; State Key Laboratory of Crop Genetics & Germplasm Enhancement and Utilization; College of Agriculture, Nanjing Agricultural University, Nanjing 211800, China

[3]College of Information Science and Technology, Gansu Agricultural University, Lanzhou 730070, China

[*] Corresponding authors:

🖂 Fangguo Chang[*]: changfg@gsau.edu.cn

🖂 Liping Wei[*]: weilp@gsau.edu.cn

Supplementary Materials

Supplementary Tables:

Table S1 Definitions and interpretations of image-derived pod-related traits used in this study.

Table S2 Descriptive statistics and heritability estimation of image-derived traits in the NJRINP population.

Table S3 Summary of cross-year stable and BLUP-supported QTL regions associated with soybean pod-related traits and the number of annotated genes within the corresponding intervals.

Table S4 Annotated genes within the focal QTL interval on chromosome 17 associated with soybean pod size-related traits.

Table S5 Annotated genes within the BLUP-supported QTL interval on chromosome 11 associated with soybean pod shape-related variation.

Table S6 Annotated genes within the combined span of the *qS19-1*, *qV19*, and *qH19* intervals on chromosome 19 associated with soybean pod color component variation.

Table S7 Annotated genes within the combined span of the *qS3* and *qV3* intervals on chromosome 3 associated with soybean pod color component variation.

Table S8 Comparison of focal QTLs identified in the present study with corresponding QTLs reported by Chang et al. (2021) in the NJRINP population.

Supplementary Figures:

Figure S1 Deep learning-based workflow used for soybean pod image segmentation and quantitative trait extraction.

Figure S2 Phenotypic distributions of eight image-derived pod-related traits in the NJRINP population.

Figure S3 Developmental expression profiles of genes within the chromosome 17 pod size-related QTL region.

Figure S4 Developmental expression profiles of genes within the BLUP-supported *qPE11* interval associated with pod elongation.

Figure S5 Developmental expression profiles of genes within the cross-year stable *qS3* and *qV3* intervals on chromosome 3.

Figure S6 Developmental expression profiles of genes within the combined span of the *qS19-1*, *qV19*, and *qH19* intervals on chromosome 19.

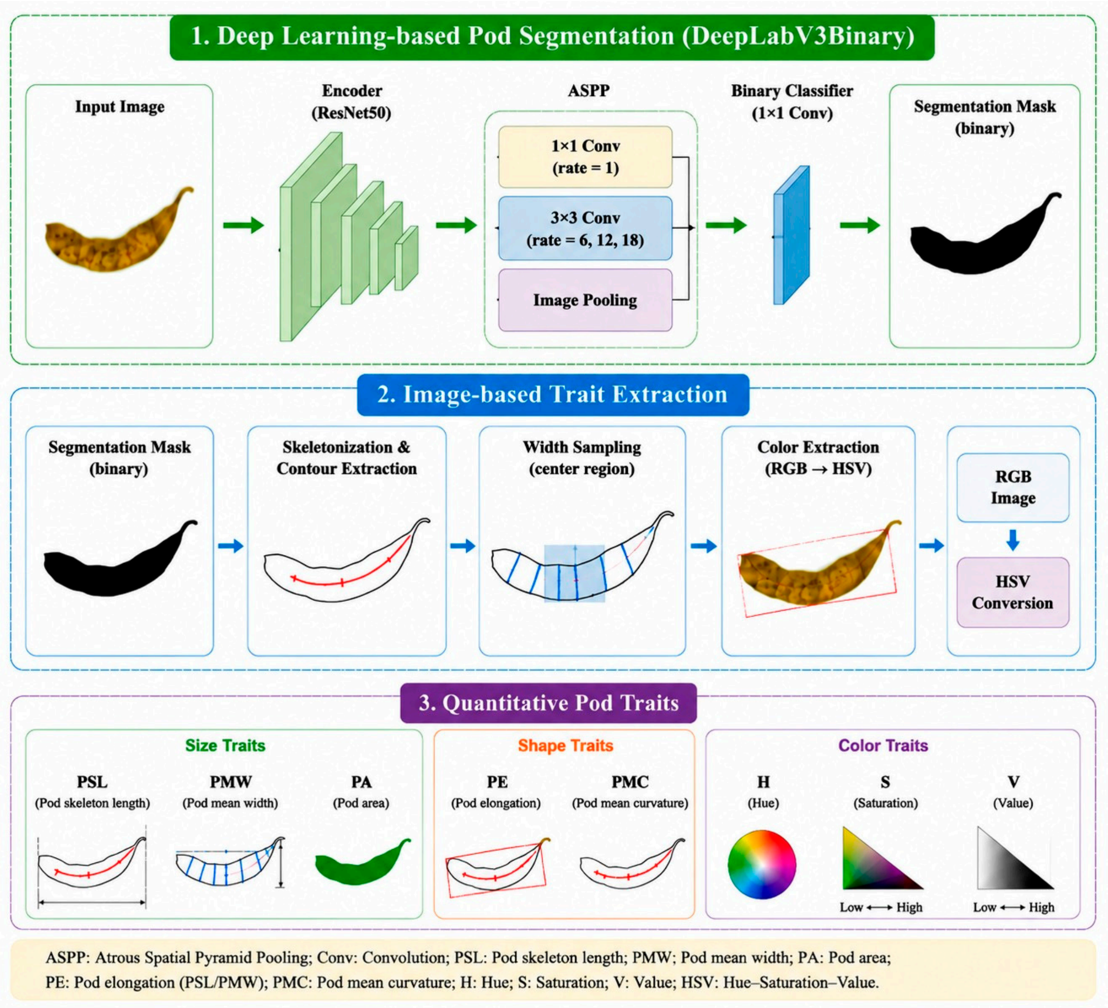

Figure S1. Deep learning-based workflow for soybean pod image segmentation and image-derived trait extraction. The upper panel shows a representative input pod image and the corresponding binary mask generated by DeepLabV3Binary. The subsequent panels illustrate skeleton and contour extraction, width sampling of the pod skeleton, RGB-to-HSV color conversion, and extraction of pod skeleton length (PSL), pod mean width (PMW), pod area (PA), pod elongation (PE = PSL/PMW), pod mean curvature (PMC), hue (H), saturation (S), and value (V). Segmentation performance on the validation set was IoU = 0.9774, Dice coefficient = 0.9885, precision = 0.9880, and recall = 0.9892 [24].

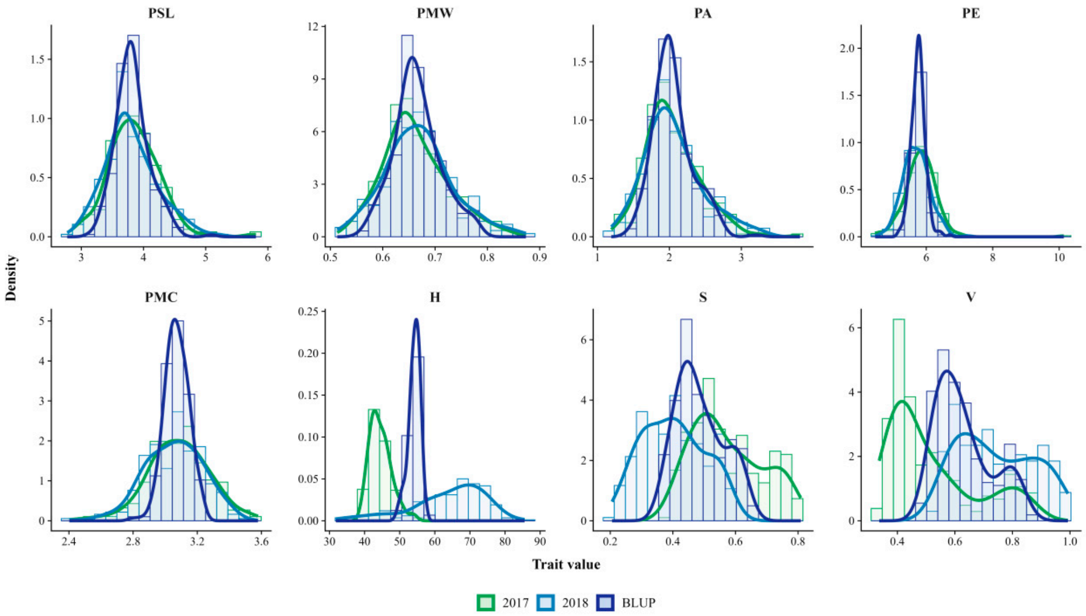

Figure S2. Phenotypic distributions of eight image-derived pod-related traits in the NJRINP population. Histograms and density curves show the 2017 (green), 2018 (light blue), and BLUP (dark blue) values for PSL, PMW, PA, PE, PMC, H, S, and V.

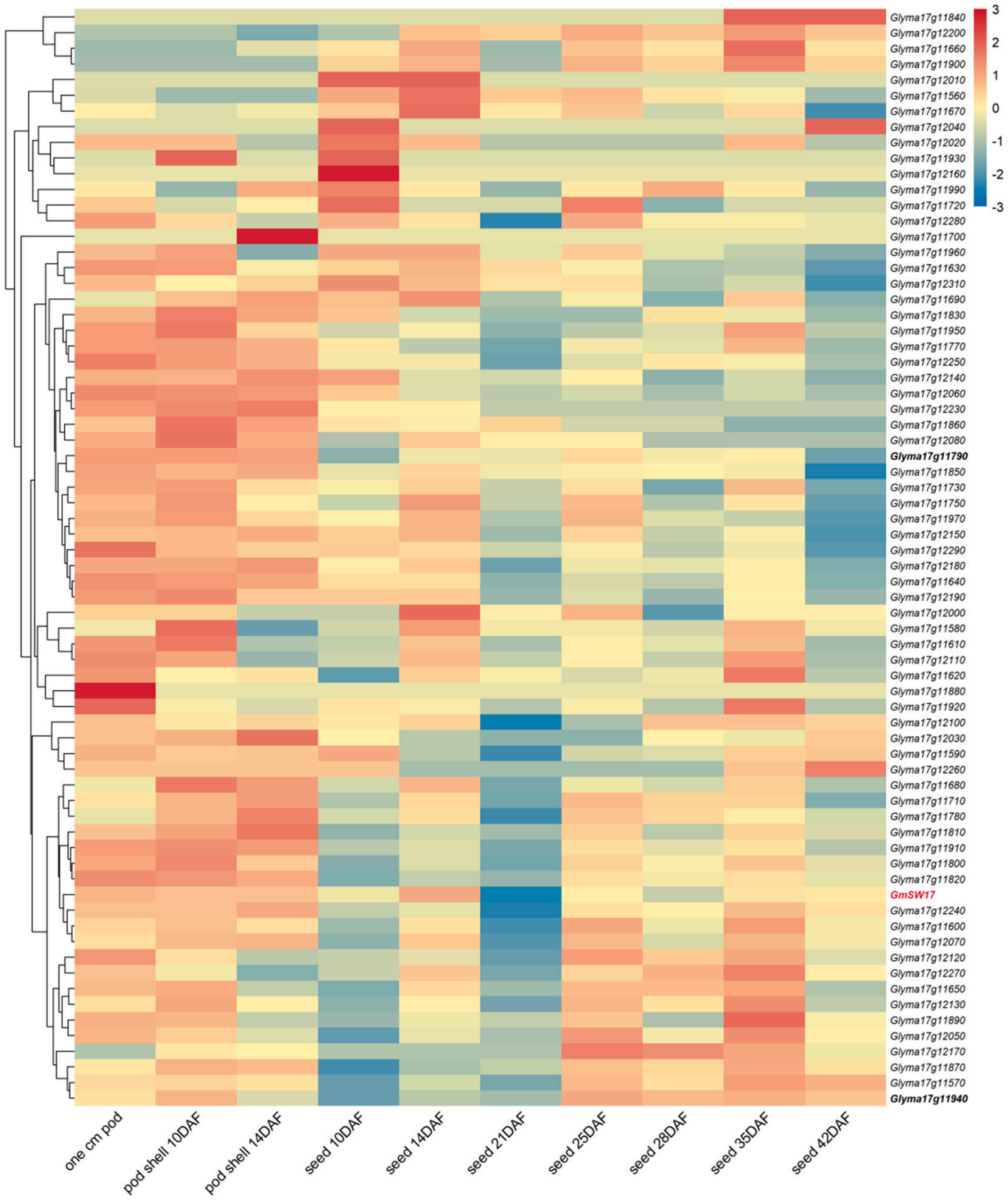

Figure S3. Developmental expression profiles of genes within the chromosome 17 pod size-related traits QTL region. The heatmap shows row-standardized expression (Z-scores) of genes in the overlapping *qPSL17-1*, *qPMW17-1*, and *qPA17* intervals during pod and seed development. *qPSL17-1* and *qPA17* were cross-year stable, whereas *qPMW17-1* was BLUP-supported. Red and blue indicate higher and lower expression, respectively. *GmSW17* (*Glyma17g11760*) is highlighted in red; *Glyma17g11790* and *Glyma17g11940* are highlighted in bold.

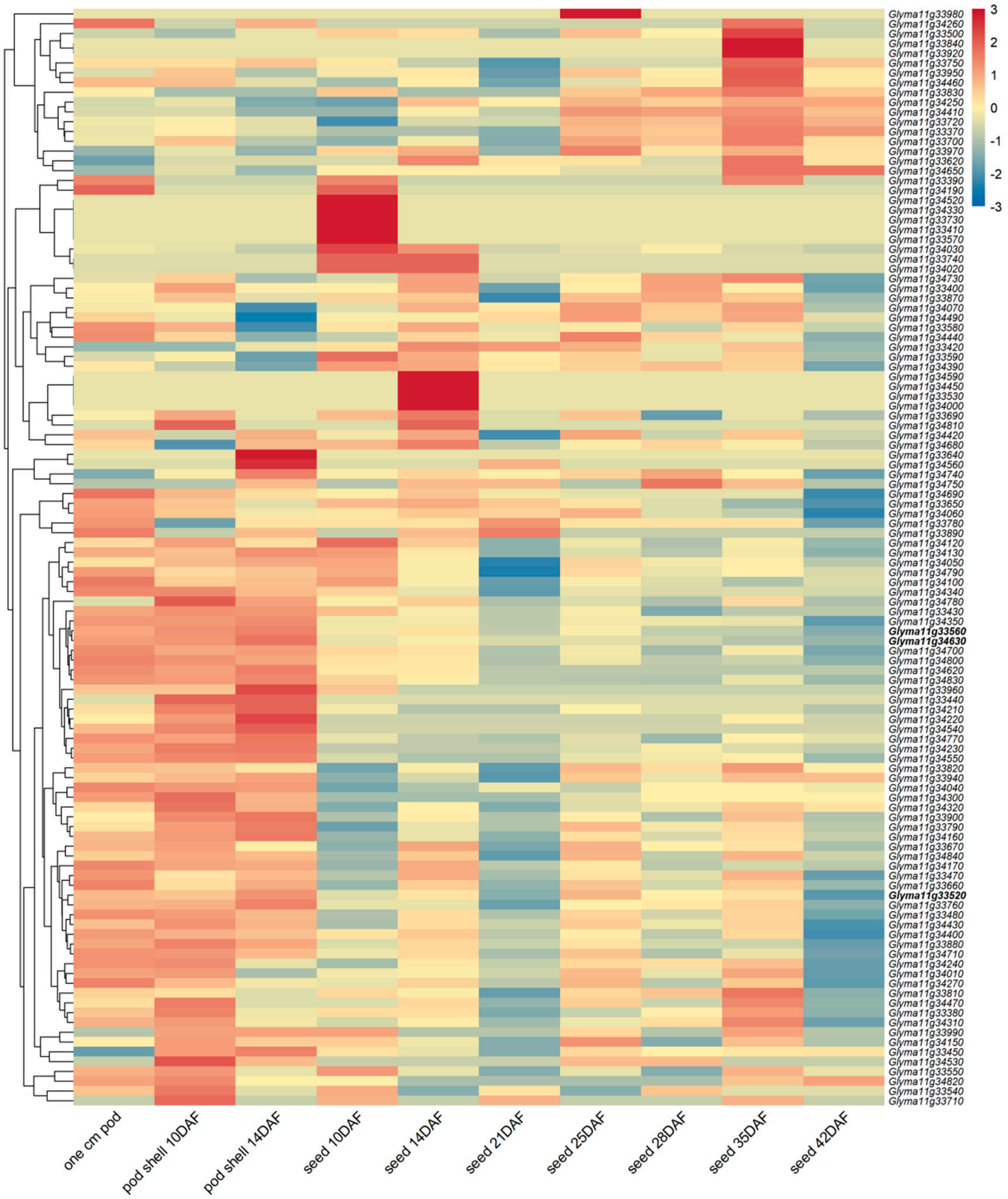

Figure S4. Developmental expression profiles of genes within the BLUP-supported *qPE11* interval associated with pod elongation. The heatmap shows row-standardized expression (Z-scores) during pod and seed development. Red and blue indicate higher and lower expression, respectively. *Glyma11g33560*, *Glyma11g33520*, and *Glyma11g34630* are highlighted in bold.

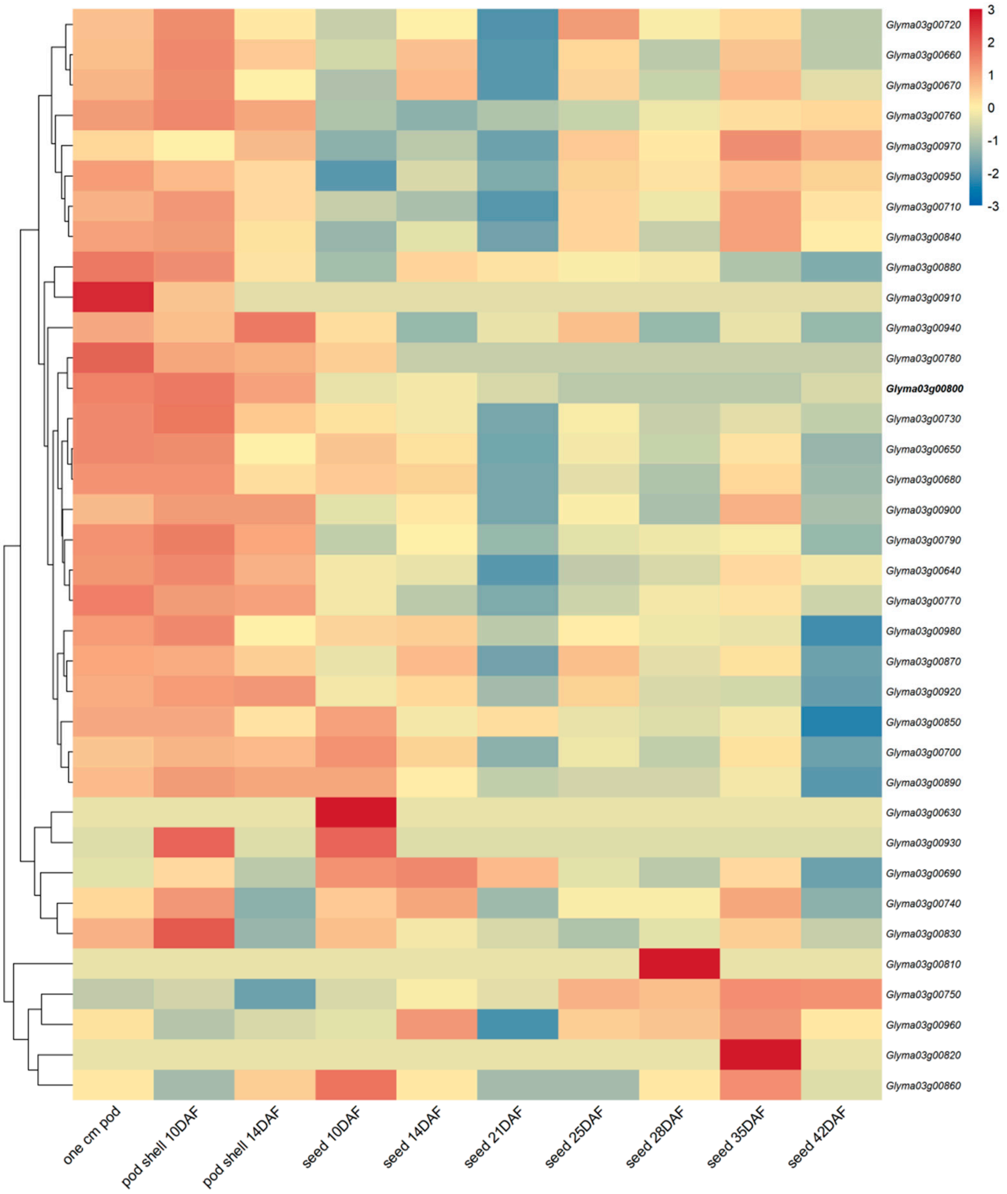

Figure S5. Developmental expression profiles of genes within the cross-year stable *qS3* and *qV3* intervals on chromosome 3. The heatmap shows row-standardized expression (Z-scores) of genes in the overlapping *qS3* and *qV3* intervals during pod and seed development. Red and blue indicate higher and lower expression, respectively. *Glyma03g00800* is highlighted in bold as an exploratory positional candidate.

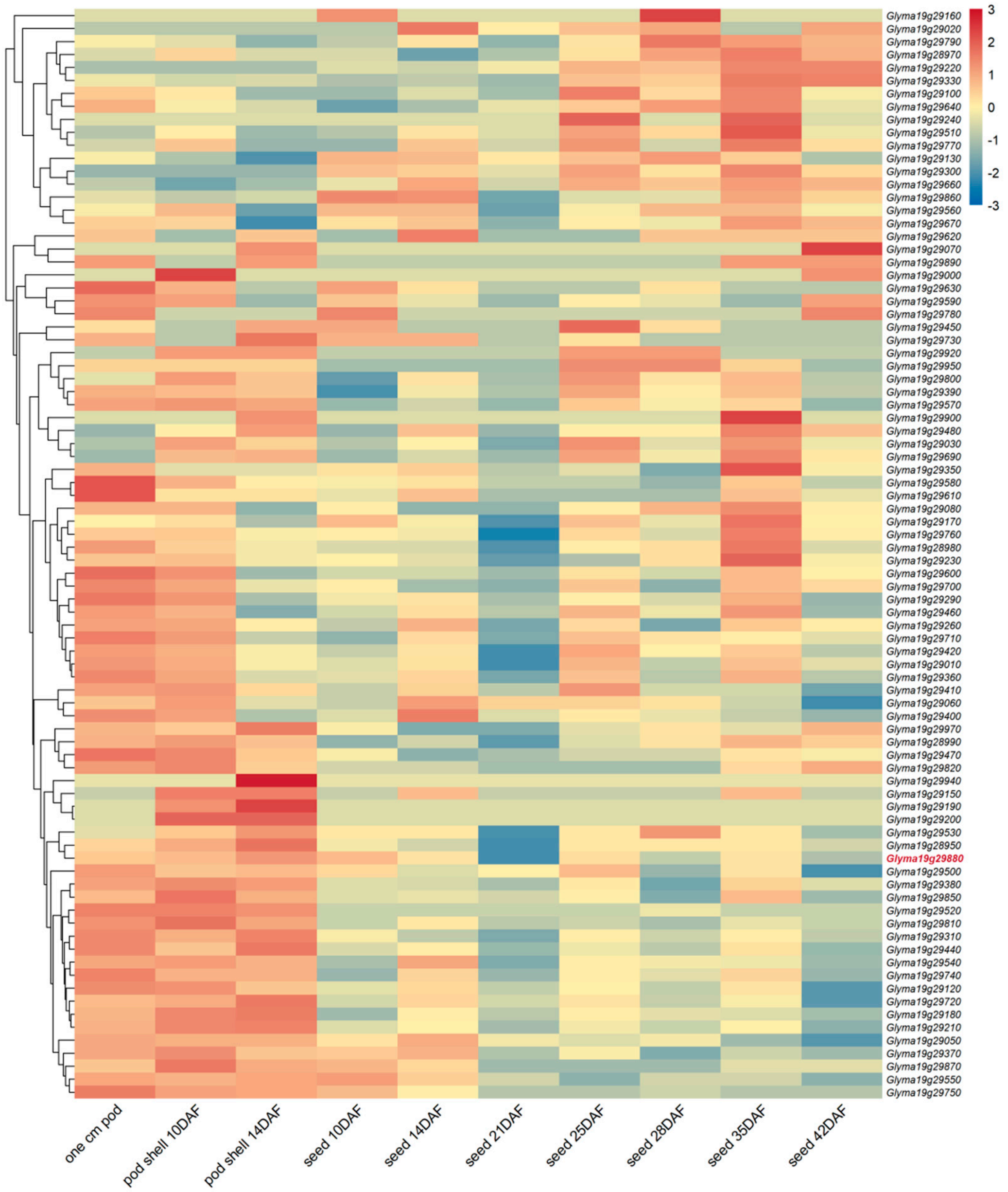

Figure S6. Developmental expression profiles of genes within the combined span of the *qS19-1*, *qV19*, and *qH19* intervals on chromosome 19. The heatmap shows row-standardized expression (Z-scores) of genes within the combined span of the *qS19-1*, *qV19*, and *qH19* intervals during pod and seed development. Red and blue indicate higher and lower expression, respectively. *Glyma19g29880* (*L1*), a validated causal gene for pod color located within *qV19*, is highlighted in red.
